# Equivalence of ELISpot Assays Demonstrated between Major HIV Network Laboratories

**DOI:** 10.1371/journal.pone.0014330

**Published:** 2010-12-14

**Authors:** Dilbinder K. Gill, Yunda Huang, Gail L. Levine, Anna Sambor, Donald K. Carter, Alicia Sato, Jakub Kopycinski, Peter Hayes, Bridget Hahn, Josephine Birungi, Tony Tarragona-Fiol, Hong Wan, Mark Randles, Andrew Raxworthy Cooper, Aloysius Ssemaganda, Lorna Clark, Pontiano Kaleebu, Steven G. Self, Richard Koup, Blake Wood, M. Juliana McElrath, Josephine H. Cox, John Hural, Jill Gilmour

**Affiliations:** 1 International AIDS Vaccine Initiative Human Immunology Laboratory, Imperial College, London, United Kingdom; 2 Statistical Center for HIV/AIDS Research and Prevention, Seattle, Washington, United States of America; 3 Foundation for the National Institutes of Health, Bethesda, Maryland, United States of America; 4 Fred Hutchinson Cancer Research Center, Seattle, Washington, United States of America; 5 Uganda Virus Research Institute, Entebbe, Uganda; 6 International AIDS Vaccine Initiative, New York, New York, United States of America; 7 Vaccine Research Center, Bethesda, Maryland, United States of America; University of Toronto, Canada

## Abstract

**Background:**

The Comprehensive T Cell Vaccine Immune Monitoring Consortium (CTC-VIMC) was created to provide standardized immunogenicity monitoring services for HIV vaccine trials. The *ex vivo* interferon-gamma (IFN-γ) ELISpot is used extensively as a primary immunogenicity assay to assess T cell-based vaccine candidates in trials for infectious diseases and cancer. Two independent, GCLP-accredited central laboratories of CTC-VIMC routinely use their own standard operating procedures (SOPs) for ELISpot within two major networks of HIV vaccine trials. Studies are imperatively needed to assess the comparability of ELISpot measurements across laboratories to benefit optimal advancement of vaccine candidates.

**Methods:**

We describe an equivalence study of the two independently qualified IFN-g ELISpot SOPs. The study design, data collection and subsequent analysis were managed by independent statisticians to avoid subjectivity. The equivalence of both response rates and positivity calls to a given stimulus was assessed based on pre-specified acceptance criteria derived from a separate pilot study.

**Findings:**

Detection of positive responses was found to be equivalent between both laboratories. The 95% C.I. on the difference in response rates, for CMV (−1.5%, 1.5%) and CEF (−0.4%, 7.8%) responses, were both contained in the pre-specified equivalence margin of interval [−15%, 15%]. The lower bound of the 95% C.I. on the proportion of concordant positivity calls for CMV (97.2%) and CEF (89.5%) were both greater than the pre-specified margin of 70%. A third CTC-VIMC central laboratory already using one of the two SOPs also showed comparability when tested in a smaller sub-study.

**Interpretation:**

The described study procedure provides a prototypical example for the comparison of bioanalytical methods in HIV vaccine and other disease fields. This study also provides valuable and unprecedented information for future vaccine candidate evaluations on the comparison and pooling of ELISpot results generated by the CTC-VIMC central core laboratories.

## Introduction

In support of the Global HIV/AIDS Vaccine Enterprise (GHAVE), the Bill & Melinda Gates Foundation funded the Collaboration for AIDS Vaccine Discovery (CAVD), an international network of 17 Vaccine Discovery Consortia with five Central Service Facilities (CSF) that provide immunology and statistical support [1], [2], [3]. As one of the CSF of the CAVD, the overall goal of the Comprehensive T Cell Vaccine Immune Monitoring Consortium (CTC-VIMC) is to provide standardized immunogenicity monitoring services in CAVD and GHAVE sponsored clinical trials of HIV vaccine candidates. To this end, the CTC-VIMC established a core of four cellular clinical immunogenicity testing laboratories, all of which are accredited to good clinical laboratory practice (GCLP) certification [4]. Core laboratories include the International AIDS Vaccine Initiative (IAVI) Human Immunology Laboratory (London, UK), the Uganda Virus Research Institute (UVRI; Entebbe, Uganda), the HIV Vaccine Trials Network Laboratory (HVTN; Seattle, US) and NVITAL, core laboratory for the Vaccine Research Center (Gaithersburg, MD).

The Enzyme-linked immunosorbent spot (ELISpot) assay is a commonly used bioanalytical method for monitoring cellular immune responses in humans and animals. While being a relatively simple assay, the ELISpot has been shown to be highly specific, sensitive with good precision and stable over time [5]. ELISpot assays were originally developed to enumerate B-cells secreting antigen-specific antibodies [6], and have since been widely used as a screening tool to assess the T- cell immunogenicity of, among others, candidate HIV vaccines [5], [7], [8], [9], [10], [11]. IFN-g secretion, as assessed by the ELISpot, occurs as a result of the recognition of cognate peptides or mitogenic stimuli by CD4 and/or CD8 T -cells. Secreted IFN-g is captured on IFN-g antibody-coated membranes and detected through subsequent recognition by further biotinylated IFN-g-specific antibodies, which in turn complex with streptavidin-conjugated enzymes that react with chromogenic substrates. The chromogenic reaction causes a spot to form where the reacting cells released their IFN-g; these spot forming units (SFUs) are then enumerated per number of stimulated Peripheral Blood Mononuclear Cells (PBMC). Typical stimulants used in such an assay are pools of overlapping synthetic peptides that correspond to sequences incorporated into vaccines. These pools consist of 8 to 15meric peptides overlapping in sequence to ensure maximal coverage of potential CD4 and CD8 epitopes.

**Figure 1:**
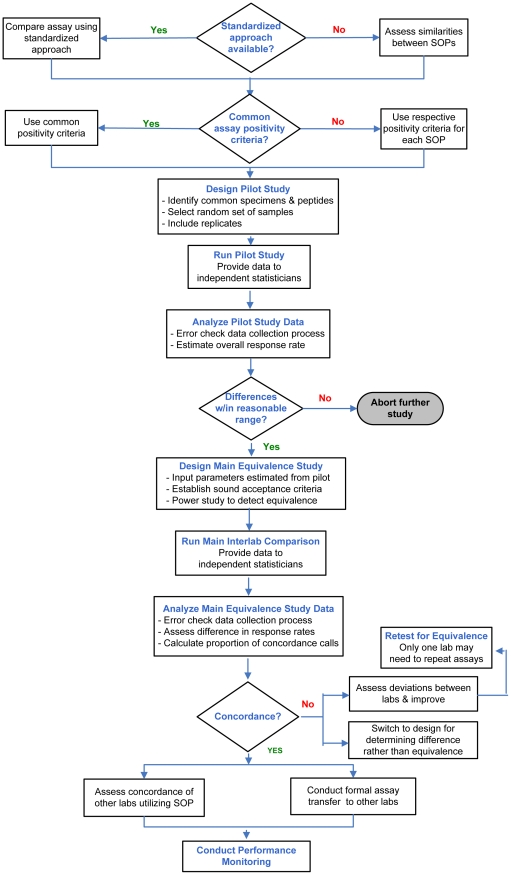
CTC-VIMC/VISC ELISpot Standardization Methodology.

Although the principal techniques underlying the assay remain constant, the use of differing SOPs for the ELISpot assay may result in variability of enumerated data between laboratories [12], [13]. Within the CTC-VIMC, both IAVI and UVRI core laboratories use the IAVI IFN-g ELISpot SOP, whereas the HVTN uses the HVTN IFN-g ELISpot SOP; both SOPs have been qualified in-house and across collaborating sites and are now routinely used to assess HIV vaccine candidates [14], [15], [16], [17]. Early plans for the CTC-VIMC were to utilize a commercially available ELISpot kit for all CAVD ELISpot tests. Unfortunately, concerns regarding reagent stability mitigated against use of these kits by the CAVD. Because significant time, effort and financial resources had been invested by IAVI and HVTN to qualify and propagate the use of SOPs across their respective laboratory networks, there was an understandable reluctance from either laboratory to use an alternative SOP when running specimens for the CAVD initiative. This study was therefore designed to generate sufficient statistical evidence to rigorously evaluate the results of the IAVI and HVTN SOPs for concordance and to guide the prospective comparison or pooling of ELISpot immunogenicity assessments across laboratories within the CAVD initiative. The CAVD Vaccine Immunology Statistical Center (VISC, Seattle, WA) assisted in the design of this comparison study and provided unbiased data management and analysis to assess this objective. The over-arching strategy for the CTC-VIMC assay comparison study, along with delineation of appropriate follow-up procedures (e.g., assay transfer and ongoing performance monitoring) is summarized in the flow chart presented in Figure 1. Use of this systematic approach resulted in the adoption of an appropriate study design. With a common set of specimens and centrally prepared stimuli and controls, the findings from this evaluation have justified the continued use of the two different SOPs within the core laboratories of the CTC-VIMC.

Because the ELISpot assay readout is often dichotomized into positive or negative responses based on pre-specified positivity criteria, this study focuses on the comparison of the two IFN-γ ELISpot SOPs with respect to the percentage of positive responses (i.e., response rates) from the tested samples and positivity call of each individual sample. Acceptance criteria on the equivalence of response rates and positivity calls were pre-specified *prior* to data analysis, thereby avoiding subjectivity of study results and inferred conclusions.

Furthermore, it was crucial to assess the false positive rates of the ELISpot assay, in addition to comparing the distribution of both background (i.e., responses from negative control wells with no antigen stimulation) and background-subtracted responses from antigen-stimulated wells. Responses to a Gag peptide pool from HIV-negative samples were used to assess the false positive rate of each assay. These assessments not only characterize the properties of the assay, but can also be used to identify sources of disagreement, if any, in the assay results.

To inform the design of the inter-laboratory comparison study, a pilot study using a small set of common samples and peptides was conducted at the IAVI and HVTN laboratories. Encouraged by evidence of concordance from data collected from the pilot study, the inter-laboratory comparison study was then designed and conducted with the appropriate sample size required to achieve statistical power in establishing equivalence between the dichotomized outcomes of the two assays.

## Materials and Methods

### Ethics Statement

All specimens provided to the laboratories were anonymized and could not be traced by to original donors. Donors provided written informed consent and study protocols had been reviewed and approved by the appropriate local Institutional Review Boards: the WP Blood Transfusion Service, Johannesburg, SA; the Seattle HIV Vaccine Trials Unit, Seattle USA; Duke University, Durham, USA; and BRT Laboratories, Baltimore USA.

### Samples

PBMC samples isolated from HIV-1 seronegative individuals with previously-characterized IFN-γ ELISpot responses to CMV pp65 peptides were selected by VISC to give evenly distributed ELISpot responses to test the low-mid dynamic range of the assay. PBMC were provided by IAVI from blood packs obtained from the South African National Blood Transfusion Services, the HVTN repository and the CTC-VIMC Proficiency Testing Core (PTC) PBMC Repository at Duke University Human Vaccine Institute, and from SeraCare Biosciences, Gaithersburg, MD. At each of these laboratories PBMC were isolated within eight hours of collection using Ficoll gradient centrifugation. Upon isolation, PBMC were frozen in a controlled stepwise manner and stored in the vapor phase liquid nitrogen. Three of the four laboratories used freeze media containing 90% FBS and 10% DMSO and SeraCare used 22% FBS, 7.5% DMSO and 70.5% RPMI (Table S1). To ensure the cold chain was maintained all freezers were constantly monitored for fluctuations in temperature. Shipments of PBMC were conducted using dry shippers (Taylor Wharton, MVE) allowing samples to be shipped to collaborating laboratories in vapor phase.

### Peptides

Peptide pools used in the study were: a pool of 32 8–10mer peptides representing immunodominant CD8+ T-cell epitopes within cytomegalovirus, Epstein Barr virus and influenza (CEF [18]; a pool of 138 15-mer peptides overlapping by 11 amino acids spanning the entire human cytomegalovirus (CMV) pp65 protein and an IAVI HIV-1 clade A Gag pool (Gag) also comprised of 90 15-mer peptides overlapping by 11 amino acids (Anaspec Inc, San Jose, CA). Peptides were used at a final concentration of 1.5 µg/mL. Phytohemagglutinin (PHA; Sigma, Dorset, UK) was used as a positive control at a final concentration of 10 µg/mL and 0.045% (final concentration) DMSO (v/v) in PBS was used as a Mock (negative control). Peptides were prepared centrally at 100× final concentration to ensure that when each site diluted their peptides to working concentration, a potential source of variation was reduced.

### ELISpot Assay

IAVI and HVTN independently developed their in-house ELISpot assays using different cell counters and ELISpot readers. Furthermore, each required the use of their own SOPs for cell thawing, counting and ELISpot. Both ELISpot SOPs employed the same anti-IFN-γ capture (1-D1K), biotinylated anti IFN-γ detection (7-B6-1) monoclonal antibodies (Mabtech, Nacka, Sweden) as well as Immobilon-P membrane ELISpot plates. Different ELISpot readers were used; HVTN used white plates (Millipore; MSIPS4W10) compatible with the CTL reader (Cellular Technologies, Cleveland, Ohio), while IAVI (and UVRI) used clear plates (Millipore; MAIPS4510) for spot enumeration by the AID ELISpot reader (AutoImmun Diagnostika, Germany). IAVI had previously compared the use of pre-coated versus self-coated ELISpot plates and found no significant difference in performance between the two [15]. For this study pre-coated plates were used exclusively by IAVI and self-coated plates were used by HVTN.

### IAVI Method Overview

Mabtech pre-coated IFN-γ ELISpot 96-well plates were washed 3 times in 200 µl PBS (Sigma, Dorset, UK) per well prior to blocking with 200 µL R10 media (RPMI 1640) supplemented with 10% (v/v) foetal bovine serum (FBS) 2 mM L-glutamine, 100 units penicillin, 0.1 mg/mL streptomycin, 10 mM HEPES buffer and 1 mM sodium pyruvate (all from Sigma) and incubated at 37°C for at least 2 hours. Cryopreserved PBMC were thawed, washed and resuspended in 5 mL R20 (R10 with 20% FBS) and incubated overnight in a humidified incubator at 37°C with 5% CO_2_ in air. On the day of assay, cells were counted (Vi-cell counter, Beckman Coulter) and resuspended in R10 at 4×10^6^ viable cells/mL. Samples with viability of less than 80% following overnight incubation were discarded and a fresh vial tested. Blocking R10 media was decanted and 100 µL of peptide (1.5 µg/mL final concentration), PHA or Mock were added followed by 50 µL of cells to give a density of 200,000 cells/well. Plates were incubated as above for 16–24 hours.

Plates were subsequently washed manually, once with 200 µL 0.05% (v/v) PBS/tween, then a further 5 times in 0.05% PBS/tween using a M384 Atlas automated plate washer (Titertek; Biological Instrumentation Services Ltd, Kirkham UK). All subsequent washes were automated. 100 µL of 0.22 µm filtered biotinylated anti IFN-γ 7-B6-1 monoclonal antibody were added at 1 µg/mL in 0.5% BSA/PBS. Plates were incubated for a further 2–4 hours at room temperature, washed 6 times with 200 µL per well with 0.05% PBS/tween, and incubated with 100 µL of avidin-biotin peroxidise complex (ABC complex; Vector labs) for 1 hour at room temperature. Plates were washed 3 times with 200 µL 0.05% PBS/tween followed by a further 3 washes with PBS prior to addition of 100 µL 3-Amino-9-ethylcarbazole (AEC) chromagen (Sigma) for 4 minutes before the reaction was stopped by rinsing under running tap water. The protective plastic backing was removed immediately and plates were left to dry overnight in the dark. SFU were enumerated using an automated AID ELISpot reader (AutoImmun Diagnostika, Germany). The pass/fail criteria set by the IAVI protocol states that the Mock wells should have less than 10 spots per well and those wells containing only R10 (no cells) should have less than 5 spots per well. For positive controls (PHA) there should be greater than 10 spots per well. If any of these criteria were not met, then the plate was failed and repeated.

### HVTN Method Overview

The HVTN method is based on the validated Merck ELISpot assay [16], 96-well hydrophobic polyvinylidene difluoride-backed plates (Millipore, Bedford, MA, US) were coated with anti-IFN-γ monoclonal antibody 1-D1K at a concentration of 10 µg/mL in PBS, overnight at 4°C. On the following day the plates were washed 4 times with 250 µL of PBS per well. 200 µL of R10 (RPMI supplemented with 10% FBS v/v (Gemini Bio-products), 2 mM L-Glutamine; 25 mM HEPES; 5 units Penicillin streptomycin (all Gibco BRL Life Technologies, Carlsbad, CA, US) were added to each well and incubated at 37°C, 5% CO_2_ for at least 2 hours. Cryopreserved PBMC were thawed, washed and resuspended in 5 mL R20 (R10 with 20% FBS) and incubated overnight in a humidified incubator at 37°C with 5% CO_2_ in air. On the day of assay set up, cell count and viability were determined using a Guava Cell Counter. Samples with <66% viability were discarded. Cells were re-suspended at 2×10^6^ cells/mL. 100 µL of cells (200,000 cells per well) and 25 µL of peptide (1.5 µg/mL final concentration), Mock or PHA were added and plates incubated at 37°C, 5% CO_2_ for 18–22 hours. The Mock normally used by HVTN is R10 (with no DMSO), but for the purpose of this study blinded stimuli included 0.45% DMSO in PBS.

Immediately prior to the end of incubation, biotinylated mouse anti-human IFN-γ 7-B6-1 solution was prepared to 1 µg/mL in 0.5% BSA/PBS diluent. Plates were washed seven times with 250 µL per well of 0.05% PBS/tween using an automated Elx405 plate washer (BIOTEK Instruments Inc, Winooski VT, US) after which 100 µL of biotinylated antibody were added and plates left at room temperature for 2–3 hours.

Following 4 washes with 250 µL/well with 0.05% PBS/tween, 100 µL of Alkaline Phosphotase-conjugated anti-biotin antibody (AP-anti biotin; diluted 1∶750 in 0.5% BSA/PBS; Vector Laboratories, Burlingame, CA, US) were added and incubated for 2–3 hours at room temperature. Plates were washed 4 times with 250 µl per well with 0.05% PBS/tween. Finally, 100 µL of BCIP/NBT (pre-filtered through 47mm Whatman filter paper; Pierce, Rockford, IL, US) were added for 7 minutes before the reaction was stopped by rinsing the plate three times with 250 µL per well of deionised water. The blue colored spots formed by IFN-γ -secreting cells were counted with an automated CTL ImmunoSpot plate reader (Cellular Technologies, Cleveland, Ohio).

The pass/fail criteria set by the HVTN protocol states that the average of the Mock wells should have less than 20 spots per well and those wells containing only R10 (no cells) should have an average of less than 6 spots. For positive controls (PHA) there should be greater than 400 spots per well. If any of these criteria are not met, then the plate is deemed to have failed and is repeated.

Potentially significant differences in method are detailed in Table 1.

**Table 1:** Differences between SOPs.

	IAVI SOP	HVTN SOP
**Plate Preparation**	Plates pre-coated with primary antibody MABTECH 1-DIK	Self-coated with primary antibody MABTECH 1-DIK
* **PBMC Counting**	Vi-Cell	Guava PCA
**PBMC Concentration**	Added 50 µL of 4.0×10^6^/mL (200,000/well final)	Added 100 µL of 2m PBMC/mL (200,000/well final)
**Substrate**	AEC	BCIP/NBT
**Spot enumeration**	AID Reader	CTL reader
**Pass Criteria**	PHA control, >10 spots per well; Mock negative control, <10 spots per well R10/CEF only <5 spots per well	PHA control, mean of 3 wells >400 spots per well, Mock negative control <20 spots per well R10 only wells, average of <6 spots per well

*Median cell recovery 74% (93% viable) at HVTN and 77% (95% viable) at IAVI.

### Study Design

Existing data were not available to help to design an efficient design for the proposed inter-laboratory equivalence study. We therefore conducted a pilot study using centrally prepared peptide pools and 30 specimen samples selected by VISC based on background-subtracted responses to CMV in the range of 0–1500 SFU /10∧6 PBMC. Both IAVI and HVTN measured responses of these 30 specimens to CMV, CEF and Gag and repeated the assay twice within their own laboratories. Data collected were used to estimate parameters needed for the design of the inter-laboratory comparison study, such as the overall response rate, the difference in response rates and the proportion of positivity calls. Data from each laboratory were selected to estimate inter-laboratory difference and duplicates within each laboratory were used to estimate the intra-laboratory difference and served as a basic assumption in deriving acceptance criteria of equivalence for the inter-laboratory comparison. Based on responses to CMV, CEF and Gag from the pilot study, a total of 155 samples from the specimen repositories were randomly selected for testing in the inter-laboratory comparison study. As data derived from the 30 pilot study samples were used to derive the acceptance criteria of equivalence, these were re-tested in the inter-laboratory comparison study to avoid the risk of over-estimating the concordance measures. Antigen responses to CMV, CEF and Gag were assessed separately in these evaluations.

A specific plate layout was adopted by both laboratories in order to reduce the impact of plate layouts on the comparison and to fulfil pass/fail and quality control criteria for both laboratories. To this end, antigen and control wells were plated in triplicate with two rows of triplicate Mock wells to incorporate IAVI's quadruplicate pass/fail criteria. For robust assessment of technique in each laboratory, all stimuli and pre-characterized PBMC were blinded. One set of instructions designed to complement both SOPs was included to ensure that plate layouts and procedures were carried out in a specified manner.

Considering the extensive experience of the UVRI laboratory with the IAVI SOP [15], the inter-laboratory reproducibility of the ELISpot assay between IAVI, HVTN and UVRI was assessed in a smaller sub-study which included 28 of the original 30 samples from the pilot.

### Statistical Analysis

Unreliable plates/samples from each lab were filtered out based on each lab's own pass/fail criteria. An assay result from the IAVI lab was included in the analysis if the mean response from Mock was <55/million PBMC. A result from the HVTN lab was included if the mean response from Mock was ≤100/million PBMC, the mean response from positive control wells was ≥2,000/million PBMC, and the variance of the three replicates divided by (median+1) was <25.

The proportion of responders (i.e., response rate) was determined based on each lab's own positivity criteria. An IAVI sample was positive for a given antigen if the mean response was >4× background (or greater than 0 if the background was 0), the coefficient of variation across the wells was <70%, and the background subtracted SFU was >38 SFU per million PBMC. The HVTN adopts positivity criteria described by Moodie et al. 2006 [19]. Because responses were examined separately, no multiplicity adjustment for multiple antigens was made in determining the positivity of responses to CMV, CEF or Gag.

The adjusted Wald interval for difference of proportions with matched pairs [20] was used to establish equivalence based on the 95% confidence interval (CI) between the difference in response rates being contained in the −15% and 15% interval. To evaluate the proportion of concordant positive responses, score-based CIs for proportions were employed. Equivalence was established if the lower bound of the 95% confidence interval on the proportion of concordant positive responses was greater than or equal to 70%.

Comparison of the background and background-subtracted responses were displayed with boxplots where the box indicates the median and interquartile range; whiskers extend to the furthest point within 1.5 times the interquartile range from the upper or lower quartile. Wilcoxon signed rank tests were used to compare the rank ordering of the responses. The Concordance Correlation Coefficient (CCC; [21]) was calculated to assess agreement and to identify any sources of disagreement of background and background-subtracted responses between results from different laboratories. The CCC is a combined measure of precision and accuracy that measures deviation from the 45-degree identity line. A concordance coefficient with value of 1 indicates a perfect agreement, −1 indicates a perfect disagreement, and 0 indicates no agreement.

Raw data from both laboratories were submitted to VISC via a secure web upload to the Atlas Portal (https://atlas.scharp.org).

## Results

### Pilot Study and Design of the Inter-Laboratory Comparison

Data from the pilot study showed that inter-lab differences in response rates were 0% for both CMV and CEF responses while the width of the 95% CI varied due to different numbers of samples being filtered for each stimulus. Intra-lab differences of the response rates between duplicate runs were in the range of 0% to 6.7% with the width of the 95% CI all being smaller than 30%. The 95% lower bound of the observed proportion of concordant pairs was in the range of 70% to 89% for inter-lab differences and 66% to 89% for intra-lab differences. See Table 2 for details. In addition, response rates of the 30 samples from the pilot were observed to be in the upper range of 73% to 83% (data not shown). These statistics observed in the pilot study served as a reference for the true values of the parameters in the sample size calculations and a gauge in determining the pre-specified acceptance criteria of equivalence for the inter-laboratory comparison.

**Table 2:** Statistics achieved in the pilot study.

	Observed differences in response rates (95% C.I.)			95% lower bound of the observed proportion of concordant pairs		
Stimuli	Inter-lab	Intra–lab 1	Intra–lab 2	Inter-lab	Intra–lab 1	Intra–lab 2
CMV	0% (6%,6%)	0% (6%, 6%)	3.3% (12%, 5%)	89%	89%	83%
CEF	0% (14%, 14%)	0% (11%, 11%)	6.7% (24%, 5%)	70%	79%	66%
Gag	0% (6%, 6%)	0% (6%, 6%)	0% (6%, 6%)	89%	83%	89%

Given the preliminary evidence of comparability between the two SOPs above, the design of the inter-laboratory comparison study proceeded. The acceptance criteria on the difference in the response rates and the proportion of concordant calls were set as the 95% CI being within the interval of [−15%, 15%] and the 95% confidence limit being greater than 70%, respectively. Based on these acceptance criteria, power calculations were conducted assuming the true response rate of 70% and 80% with sample sizes of 90, 120 and 150. 5000 datasets were simulated to assess the empirical statistical power with both acceptance criteria satisfied with a type I error rate of 0.05. With a sample size of 150, reasonable power (∼80%) can be achieved if the true proportion of concordance is at least 90% when the true difference in response rates is no more than 6%. In total, 155 samples were selected for the inter-laboratory comparison study allowing for a 3% possible assay failure rate as observed in the pilot study.

### Inter-laboratory Comparison

Data from the inter-laboratory comparison study demonstrated that both response rates and positivity calls passed the pre-specified acceptance criteria for equivalence (Table 3). Specifically, for CMV responses, the 95% C.I. on the difference in response rates was (−1.5%, 1.5%) and the lower bound of the 95% score confidence interval on the proportion of concordant positivity calls was 97.2%. For CEF responses, the 95% C.I. on the difference in response rates was (−0.4%, 7.8%); the lower bound of the 95% score confidence interval on the proportion of concordant positivity calls was 89.5%. The 95% C.I. on the difference in response rates was contained in the pre-specified equivalence interval of [−15%, 15%] for both CMV and CEF responses; the lower bound of the 95% confidence interval on the proportion of concordant positivity calls was also greater than the pre-specified equivalence margin of 70%. Note that not all 155 samples contributed evaluable data: based on pre-specified filtering criteria, 5 CMV responses, 5 CEF responses and 5 HIV A Gag responses were excluded in the IAVI dataset; 15 CMV responses, 14 CEF responses and 14 Gag responses were excluded in the HVTN dataset. Consequently, the numbers of evaluable samples for each antigen from each lab are smaller than the total number of samples (n = 155) that were tested at each laboratory.

**Table 3:** Results on the comparison of response rates and positivity calls in the inter-laboratory comparison study.

Antigen	IAVI response rate	HVTN response rate	Difference in response rate (95% CI)	Concordance (LB 95% CI)
CMV	98/146 = 67.1% (59.1%, 74.2%)	87/136 = 64.0% (55.6%, 71.6%)	0/131 = 0.0% (−1.5%, 1.5%)	131/131 = 100% (97.2%, 100%)
CEF	118/146 = 80.8% (73.7%, 96.4%)	114/137 = 83.2% (76.1%, 88.5%)	5/132 = 3.8% (−0.4%, 7.8%)	125/132 = 94.7% (89.5%, 97.4%)
Gag	6/146 = 4.1% (1.9%, 8.7%)	7/137 = 5.1% (2.5%, 10.2%)	3/132 = 2.3% (−1.9%, 6.4%)	125/132 = 94.7% (89.5%, 97.4%)

**Figure 2:**
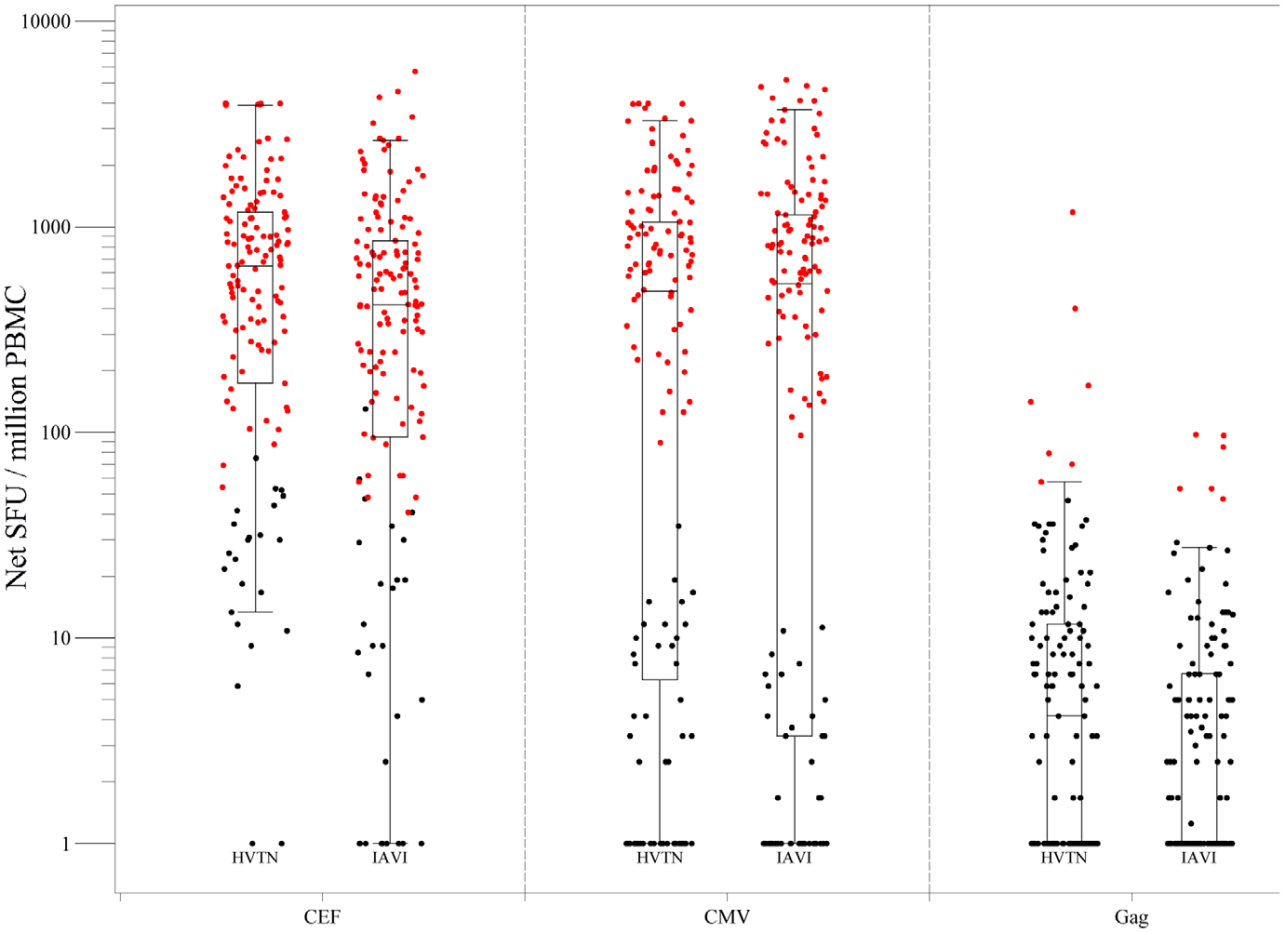
Boxplots of net IFN-g ELISpot responses from the inter-laboratory comparison study. Boxes represent the inter-quartile range of 25–75^th^ percentiles, the whiskers extend to the most extreme data point which is no more than 1.5 times the inter-quartile range from the box. The color of the dots indicate the positivity of the actual responses (red for positive and black for negative) determined by the positivity criteria described in section 4.5. SFU = spot forming units.

**Figure 3:**
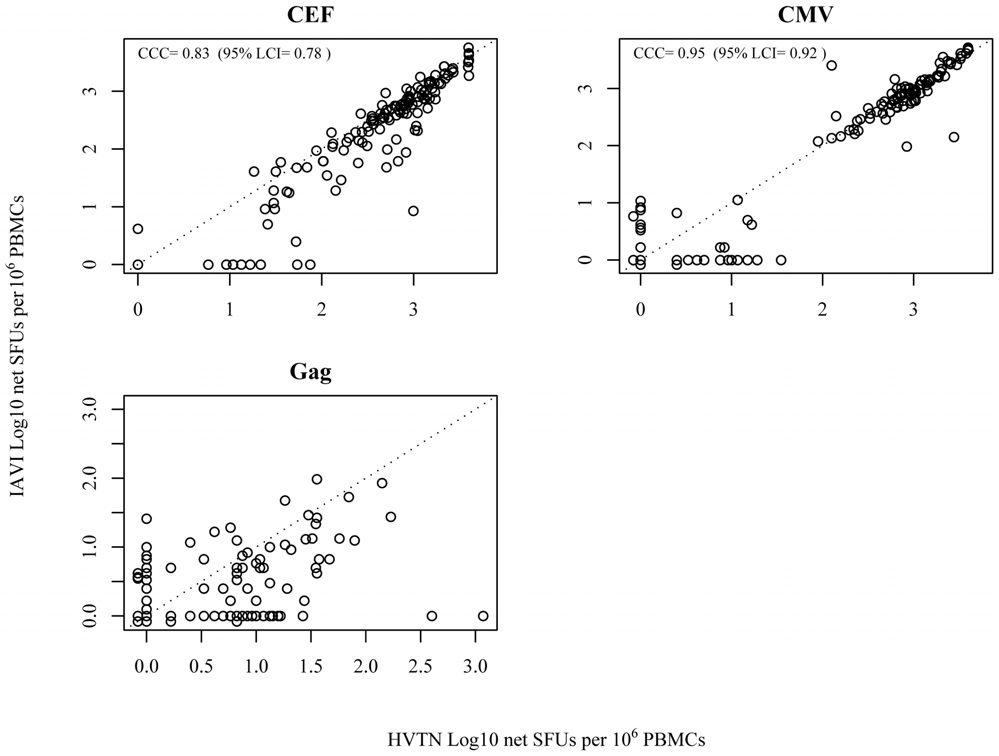
Scatter plot of all net CMV IFN-g ELISpot responses between the IAVI & HVTN. The dotted line is an identity line indicating perfect concordance. The CCC's were calculated based on methods referred in section 4.5. SFU = spot forming units.

This study demonstrates the ability of two central laboratories, IAVI and HVTN, using their respective validated IFN-γ ELISpot SOPs, to produce highly comparable results. As detailed in Table 3, the response rates from the two labs were: 98/146 (67.1%) vs. 87/136 (64%) for CMV and 118/146 (80.8%) vs. 114/137 (83.2%) for CEF (IAVI and HVTN respectively). In addition, very low false positive rates assessed on Gag were achieved in both laboratories with 6/146 (4.1%) from the IAVI laboratory and 7/137 (5.1%) from the HVTN laboratory. Secondary comparisons on the quantitative responses show that magnitudes of the raw CMV and CEF response were similar between the two laboratories. As shown in Figure 2, the median CMV responses were 542 SFUs/10^6^ PBMC (range: 0 to 5193) and 513 SFUs/10^6^ PBMC (range: 3 to 4000) for IAVI and HVTN respectively; the median CEF responses were 423 SFUs/10^6^ PBMC (range: 0 to 5713) and 646 SFUs/10^6^ PBMC (range: 15 to 4000) for IAVI and HVTN respectively. In addition, the rank ordering of the net responses were tested comparable between the two laboratories for CMV and CEF (p = 0.74 and 0.93, respectively). Overall, magnitudes of the net CMV and CEF responses between IAVI and HVTN also showed high concordance with CCC of 0.95 for all responses and CCC of 0.8 for positive responders; Figure 3).

#### Comparison to a Third Laboratory

**Figure 4:**
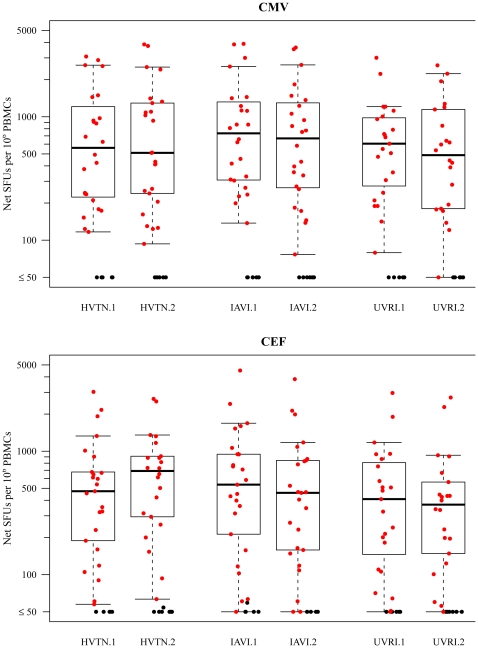
Boxplots of net CMV IFN-g ELISpot responses from the 30 pilot study samples measured in duplicates within each of the 3 laboratories. Inter- and intra-laboratory analysis was performed between the UVRI, IAVI Core laboratory and HVTN laboratory results. 28 of the 30 pilot study samples were also tested by UVRI. Boxes represent the interquartile range of 25–75^th^percentile; bars represent the 95^th^ percentile of IFN-g ELISpot responses. SFU = spot forming units.

Although the similarity of responses between the IAVI and UVRI had been previously established through proficiency testing (15), the availability replicate samples from the pilot study enabled a comparison of data generated by UVRI to that from IAVI and HVTN. Due to the small sample size of this comparison, no formal hypotheses testing procedures were carried out for these data. Nevertheless, the observed response rates and response magnitudes across the three labs showed encouraging comparability. Specifically, the response rates to CMV, CEF and Gag between the three laboratories were; for CMV 80%, 80% and 79%; for CEF 83%, 83%, 79%; for Gag 0%, 0% and 3% (for HVTN, IAVI and UVRI, respectively). In addition, Figure 4 shows boxplots of net responses to CMV and CEF for 30 samples (28 of 30 for UVRI) tested on two runs each at HVTN, IAVI and UVRI laboratories. Responses to Gag were all below 50 SFUs/10^6^ PBMC (data not shown). The concordance correlation coefficients between IAVI and UVRI for CMV and CEF are 0.95 and 0.8, respectively (data not shown).

## Discussion

The IFN-γ ELISpot assay has been used routinely to evaluate immune responses to vaccine candidates for HIV, other infectious diseases and cancer. It is often used as a first line-screening assay due to speed, ease of use, sensitivity and cost effectiveness. Indeed, the increased use of automated ELISpot readers [22] and plate washers have allowed the semi-automated quantification of antigen-specific cells [23]. With achievement of the pre-specified criteria, this study has determined that different SOPs developed for the same assay can yield comparable results. Moreover, what were considered major differences in methodology did not affect the overall sensitivity of the assay. It was also speculated at the study's outset that the two cell counters might be a source of discordant responses, but cell recoveries and viabilities were comparable for both laboratories.

This comparability was indeed a fortuitous outcome; had equivalence not been demonstrated, the CTC-VIMC would have adopted one SOP for use by the four core laboratories when performing immunogenicity assays for the CAVD. Further, these positive findings have supported an assessment of the comparability of ELISpot results from a third central laboratory (UVRI), follow-up transfer of one of the SOPs to a fourth central laboratory (NVITAL), and the implementation of a longitudinal performance monitoring program. Previous studies have raised concerns regarding variability in ELISpot assays between laboratories [12]. However, when transferred using stringent training programs, in addition to standardized equipment and reagents, assay reproducibility has been demonstrated across multiple GCLP accredited laboratories [14], [15], [17], [24] supporting the role of core laboratories.

Contributions of this study are three-fold: first, a prototypic example is provided of the process needed to conduct similar types of studies to evaluate other bioanalytical methods in the fields of vaccines and other diseases; second, the paired-data collected in such a comparison study provide information on appropriate calibration factors to apply to future studies when IFN-γ ELISpot assay results assessing the same vaccine product from the two labs are pooled; and lastly rigorous statistical evidence shows that the dichotomized IFN-γ ELISpot assay data generated from the IAVI and HVTN laboratories are comparable and assures possible comparisons of results on independent samples between these two major HIV network laboratories.

Combined with data from UVRI, our study supports the transfer of these qualified SOPs across a laboratory network and has enabled the fourth CTC-VIMC core laboratory, NVITAL, to select and complete a rigorous technology transfer of one of the ELISpot SOPs.

In implementing the GHAVE model, the CAVD forged new collaborations to leverage of the expertise of scientists devoted to HIV/AIDS vaccine research. This was especially true for the central laboratories of the CTC-VIMC whose members represent major stakeholders in HIV vaccine development: IAVI, HVTN and NIAID. With resources provided to standardize T cell assays for CAVD and GHAVE sponsored trials, our core clinical laboratories were given an unprecedented opportunity to objectively assess the equivalence of their respective ELISpot assays. What began as an effort to identify a single standardized assay for monitoring CAVD sponsored vaccine has already yielded greater impact on the HIV/AIDS vaccine field. It is now established that the results of studies with ELISpot measures performed by the IAVI or HVTN networks are suitable for comparison, and that rational conclusions can be based on such comparisons.

The CTC-VIMC central repository was able to supply PBMC obtained in sufficiently large numbers through leukapheresis. The importance of this resource can not be over emphasized: the availability of replicate samples allowed two and three way inter-laboratory comparisons, as well as subsequent technology transfer of the ELISpot assay to the fourth CTC-VIMC core laboratory. After an inter-lab assay comparability study is concluded and formal assay transfers have been completed, regular monitoring of the performance and robustness of the assay is recommended (See Figure 1). The PBMC supplied by the CTC-VIMC repository has also permitted implementation of a quality assessment program to monitor ELISpot performance over time using common reagents and a panel of specimens. Such a program of follow-up testing is designed to rapidly detect any performance deviations if they occur [13], [15], [25]. Now completing its fourth quarterly assessment, the CTC-VIMC's monitoring program has generated data that demonstrate consistent performance of the ELISpot assay overtime in all four central laboratories.

While HIV researchers have the definite advantages of a single disease focus, a somewhat common set of reagents (HIV peptides), and adequate funding, this study offers hope to other researchers who rely on the IFNγ ELISpot that in GCLP laboratories, sources of variability can in fact be controlled and systematically evaluated [13]. Other disease specific collaborations can benefit by incorporating similar design, methods and material considerations as they approach standardising their T cell immune monitoring assays.

## Supporting Information

Table S1 Details of PBMC processing and subsequent viability and recovery.
(0.03 MB PDF)
