# Supplementary material for: Equivalence of ELISpot Assays Demonstrated between Major HIV Network Laboratories
Source: PLoS One. 2010 Dec 14;5(12):e14330. doi: 10.1371/journal.pone.0014330 (PMC3001861; doi:10.1371/journal.pone.0014330)
Supplement: Table S1 — Details of PBMC processing and subsequent viability and recovery. (0.03 MB PDF) [file pone.0014330.s001.pdf]

Supplementary Table 1

Source of PBMC	Freeze media	Collection processes	HVTN	IAVI	HVTN	IAVI
			Median Viability overnight		Median recovery overnight	
Duke (n=19)	90%FBS/10% DMSO	Leukopheresis	93	95	80	90
HVTN (n=28)		Leukopheresis	94	96	75	80
CLS, IAVI (n=79)		Buffy Coat	93	95	75	75
SeraCare (n=24)	22% FBS/ 7.5% DMSO/ 70.5% RPMI	Leukopheresis	94	95	65	65
